# Efficient CT Metal Artifact Reduction Based on Fractional-Order Curvature Diffusion

**DOI:** 10.1155/2011/173748

**Published:** 2011-07-24

**Authors:** Yi Zhang, Yi-Fei Pu, Jin-Rong Hu, Yan Liu, Qing-Li Chen, Ji-Liu Zhou

**Affiliations:** ^1^College of Computer Science, Sichuan University, Chengdu 610065, China; ^2^School of Electronics and Information Engineering, Sichuan University, Chengdu 610065, China

## Abstract

We propose a novel metal artifact reduction method based on a fractional-order curvature driven diffusion model for X-ray computed tomography. Our method treats projection data with metal regions as a damaged image and uses the fractional-order curvature-driven diffusion model to recover the lost information caused by the metal region. The numerical scheme for our method is also analyzed. We use the peak signal-to-noise ratio as a reference measure. The simulation results demonstrate that our method achieves better performance than existing projection interpolation methods, including linear interpolation and total variation.

## 1. Introduction

Metal artifact reduction (MAR) is still one of the major challenges in X-ray computed tomography (CT) imaging [1–6]. For high density objects, such as a metal object, the severe attenuation of X-rays allows only a limited number of photos to reach the receiving CT array of sensors. As a result, streak artifacts appear in the reconstructed image after filtered back projection (FBP). The artifacts spread through the whole image, thereby contaminating the imaging quality.

 Since the projection data of the metal regions are much larger than ordinary tissue projection data sets, we can assume that the segments of the sinogram with the metal projection data sets are dominated by the metal component only. Based on this assumption, we can deal with projection data of metal objects as lost information, using one of the two main categories of methods: projection interpolation methods [7–12] or iterative reconstruction methods [13–17]. After theoretical analysis, MAR methods based on iterative reconstruction have better reconstruction performance than projection interpolation methods, but they often incur high computational costs and are difficult to implement in current CT imaging systems. In this paper, we concentrate on projection interpolation methods for MAR. In 1978, Lewitt and Bates used a Chebyshev polynomial to implement interpolation [7]. Kalender et al. employed linear interpolation (LI) [8], while Crawford added various assistant processes based on it [9]. Also based on linear interpolation, Gu et al. presented a more accurate metal region segmentation method using the differences in neighboring pixels [10]. Zhao et al. proposed interpolating the wavelet coefficients of the projection data [11]. To obtain a better visual effect, inpainting based on partial differential equations (PDEs) was introduced in [12, 13]. The inpainting method proposed by Gu et al. is based on Euler's elastica and curvature [12]. Duan et al. introduced a classical image inpainting method based on total variation (TV) for MAR [13]. However, the existing PDE image inpainting method cannot connect a wide inpainting region smoothly, and therefore, if wide regions exist, it does not achieve satisfactory results.

In this paper, we propose a fractional-order curvature-driven diffusion (FCDD) MAR model based on our generalized image regularization framework [18]. First, we introduce the FCDD inpainting model. Then, we give the numerical scheme for our method. After presenting our simulation, we conclude this paper.

## 2. Method

**Figure 1:**
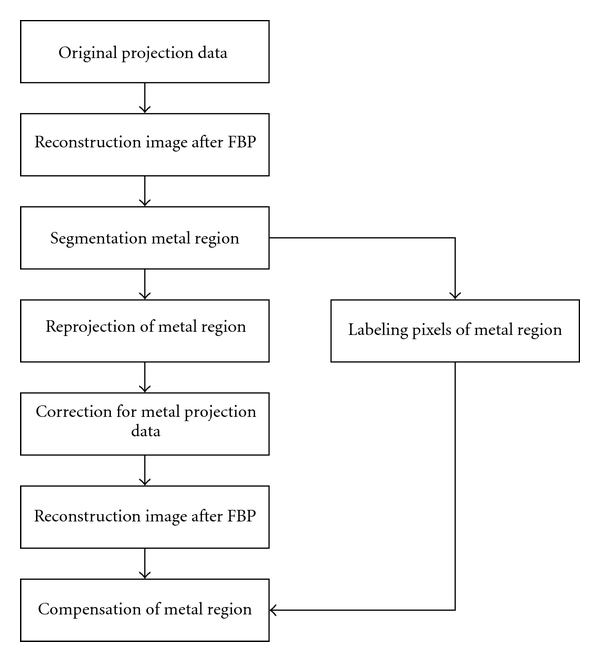
Flowchart of projection interpolation algorithms.

 The main steps in our algorithm are similar to those in conventional projection interpolation algorithms as shown in Figure 1.

 The main difference between our method and the conventional projection interpolation methods lies in the step “correction for metal projection data.” When the metal damages the original projection data in the form of a gap, conventional methods apply interpolations [7, 8] or PDE inpainting [12, 13] algorithms to restore the data gap. But these conventional methods have some drawbacks. First, the gap boundaries after inpainting lack smoothness. Second, if the gap is wide, the inpainting results do not achieve satisfactory visual effects. The proposed CT MAR method is based on FCDD that can fix these flaws. This method achieves smoother results and can also cope with the artifacts caused by wide data gaps. In this section, we first introduce the well-known TV inpainting model, and then present our FCDD model. Finally, the numerical algorithm is given for our model.

### 2.1. Review of Classic TV Inpainting Model

Assume a standard image model defined as

(1) $$u\left(x\right)=u_{0}\left(x\right)+n\left(x\right),$$

where *u*_0_ is the original image, *n* is additive noise, and *u* is the contaminated image with noise.

 Let *Ω* be the inpainting (open) domain with its boundary ∂*Ω*, and *E* an extended domain surrounding the ∂*Ω* such that ∂*Ω* lies within *E* ∪ *Ω*. The image inpainting model based on TV proposed by Chan and Shen [19] is followed here:

(2) $$\min\;J_{\lambda }\left[u\right]=\int_{E\cup \Omega }\left|\nabla u\right|dx\;dy+\frac{\lambda_{\Omega }}{2}\int_{E}{\left|u-u_{0}\right|}^{2}dx\;dy.$$

The first term is the regularizing term for inpainting damaged domains, while the second term in the energy function is the data fidelity term that can keep important features and sharp edges when noise exists. *λ*_*Ω*_ is a scale function to tune the weights of the two terms. According to variational theory, the Euler-Lagrange equation corresponding to (2) is

(3) $$-\nabla \cdot \left({\left|\nabla u\right|}^{-1}\nabla u\right)+\lambda_{\Omega }\left(u-u_{0}\right)=0$$

with the Neumann boundary condition, ∂*u*/∂*n* = 0 on ∂*Ω*, where

(4) $$\lambda_{\Omega }=\lambda \cdot 1_{E}\left(x,y\right)=\{\begin{array}{cc} \lambda , & \left(x,y\right)\in E\\ 0, & \;\text{otherwise.} \end{array}$$

This model is inspired by the classic TV denoising model [20].

### 2.2. Proposed FCDD Inpainting Model

In the classical TV inpainting model, the conductivity coefficient of the diffusion strength, which only depends on the numerical value of the isophotes, is

(5) $$F={\left|\nabla u\right|}^{-1}.$$

The geometric information of the isophotes is not considered, which is why a wide gap cannot be restored perfectly. Motivated by the methods in [21, 22], to recover from this situation, we use a new fractional-order conductivity coefficient instead of the old one:

(6) $$F=f\left(\left|\kappa^{\alpha }\right|\right){\left|\nabla^{\alpha }u\right|}^{-1},$$

where *f*(·) is a function with the following property:

(7) $$\begin{array}{cc} f\left(s\right) & =\{\begin{array}{cc} 0, & s=0\\ \infty , & s=\infty \\ \text{between}\;0\;\text{and}\;\infty , & 0<s<\infty . \end{array} \end{array}$$

If the isophote has a large fractional-order curvature, the diffusion strength is also large. In this paper, we choose *f*(*s*) = *s*. Thus, the Euler-Lagrange equation for the FCDD model is

(8) $$\begin{array}{c} \frac{\partial u}{\partial t}=\nabla \cdot \left[f\left(\left|\kappa^{\alpha }\right|\right){\left|\nabla^{\alpha }u\right|}^{-1}\nabla u\right],\;\text{in}\;D,\\ u=u_{0},\;\text{in}\;D^{c}. \end{array}$$

Here, the inpainting domain *D* is a mathematical open set, *D* ^*c*^ denotes the outer area of *D*, and *u*_0_ is the available part of the image. The fractional-order curvature *κ* ^*α*^ is defined as

(9) $$\kappa^{\alpha }=\nabla^{\alpha }\cdot \left[\nabla^{\alpha }u{\left|\nabla^{\alpha }u\right|}^{\left(-1\right)}\right].$$

### 2.3. Numerical Scheme

In this section, we apply a time marching scheme to our model. Assuming a time step size of Δ*t* and a space grid size of *h*, we let

(10) $$\begin{array}{cc} x_{i} & =ih,\;y_{j}=jh,\;\;\;i,j=0,1,\ldots ,N,\;\text{with}\;Nh=1,\\ t_{n} & =n\Delta t,\;n=0,1,\ldots \end{array}$$

The explicit scheme iterates as

(11) $$u^{n+1}=u^{n}+\Delta t\left(\Delta u\left|\kappa^{\alpha }\right|{\left|\nabla^{\alpha }u\right|}^{-1}\right).$$

First, we describe the discretization of the fractional-order gradient operator ∇^*α*^ and fractional-order Laplacian operator Δ^*α*^ using the Grümwald-Letnikov definition [23] in fractional calculus as the mask we proposed in [24, 25].

The *α*-order Grümwald-Letnikov definition-based fractional differential can be expressed as

(12) $$\begin{array}{cc} D_{\text{G-L}}^{\alpha }\;s\left(x\right) & ={\;\frac{d^{\alpha }}{{\left[d\left(x-a\right)\right]}^{\alpha }}s\left(x\right)|}_{\text{G-L}}\\ & =\underset{N\to \infty }{\lim\;}\{\frac{{\left((x-a)/N\right)}^{-\alpha }}{\Gamma \left(-\alpha \right)}\overset{N-1}{\underset{k=0}{\sum }}\frac{\Gamma \left(k-\alpha \right)}{\Gamma \left(k+1\right)}\;\\ & \;\;\;\;\;\;\times \;s\left(x-k\left(\frac{x-a}{N}\right)\right)\}, \end{array}$$

where the duration of signal *s*(*x*) is [*a*, *x*], *α* is any real number, and *s*(*x* − *k*((*x* − *a*)/*N*)) is the discrete sampling. 

If *N* is big enough with *a* = 0, the limit symbol can be dispensed with and (12) is rewritten as

(13) $$\begin{array}{cc} & {\;\frac{d^{\alpha }}{dx^{\alpha }}s\left(x\right)|}_{G\text{-}L}\\ & \;\;≅\frac{x^{-\alpha }N^{\alpha }}{\Gamma \left(-\alpha \right)}\overset{N-1}{\underset{k=0}{\sum }}\frac{\Gamma \left(k-\alpha \right)}{\Gamma \left(k-1\right)}s\times \left(x+\frac{\alpha x}{2N}-\frac{kx}{N}\right). \end{array}$$

To obtain the value of *s*(*x* + *αx*/2*N* − *kx*/*N*), we use Lagrange 3-point interpolation with *s*(*x* + *x*/*N* − *kx*/*N*), *s*(*x* − *kx*/*N*), and *s*(*x* − *x*/*N* − *kx*/*N*). Then, we obtain

(14) $$\begin{array}{cc} & \frac{d^{\alpha }}{dx^{\alpha }}s\left(x\right)\\ & \;\;≅\frac{x^{-\alpha }N^{\alpha }}{\Gamma \left(-\alpha \right)}\overset{N-1}{\underset{k=0}{\sum }}\frac{\Gamma \left(k-\alpha \right)}{\Gamma \left(k+1\right)}\\ & \;\;\;\times \left[s_{k}+\frac{\alpha }{4}\left(s_{k-1}-s_{k+1}\right)+\frac{\alpha^{2}}{8}\left(s_{k-1}-2s_{k}+s_{k+1}\right)\right]. \end{array}$$

If *k* = *n* ≤ *N* − 1, from (13), the anterior *n* + 2 approximate backward differences of the fractional partial differentials on the negative *x*- and *y*-axes, respectively, are expressed as

(15) $$\begin{array}{cc} & \frac{\partial^{\alpha }s\left(x,y\right)}{\partial x^{\alpha }}\\ & ≅\left(\frac{\alpha }{4}+\frac{\alpha^{2}}{8}\right)s\left(x+1,y\right)\\ & \;\;+\left(1-\frac{\alpha^{2}}{2}-\frac{\alpha^{3}}{8}\right)\times s\left(x,y\right)+\frac{1}{\Gamma \left(-\alpha \right)}\\ & \;\;\times \overset{n-2}{\underset{k=1}{\sum }}[\frac{\Gamma \left(k-\alpha +1\right)}{\left(k+1\right)!}\cdot \left(\frac{\alpha }{4}+\frac{\alpha^{2}}{8}\right)+\frac{\Gamma \left(k-\alpha \right)}{k!}\;\\ & \;\;\;\;\;\cdot \left(1-\frac{\alpha^{2}}{4}\right)+\frac{\Gamma \left(k-\alpha -1\right)}{\left(k-1\right)!}\cdot \left(-\frac{\alpha }{4}+\frac{\alpha^{2}}{8}\right)]\\ & \;\;\times s\left(x-k,y\right)+[\frac{\Gamma \left(n-\alpha -1\right)}{\left(n-1\right)!\Gamma \left(-\alpha \right)}\cdot \left(1-\frac{\alpha^{2}}{4}\right)\;\\ & \;\;\;\;\;\;\;\;\;+\frac{\Gamma \left(n-\alpha -2\right)}{\left(n-2\right)!\Gamma \left(-\alpha \right)}\cdot \left(-\frac{\alpha }{4}+\frac{\alpha^{2}}{8}\right)]\\ & \;\;\times s\left(x-n+1,y\right)+\frac{\Gamma \left(n-\alpha -1\right)}{\left(n-1\right)!\Gamma \left(-\alpha \right)}\\ & \;\;\cdot \left(-\frac{\alpha }{4}+\frac{\alpha^{2}}{8}\right)\times s\left(x-n,y\right),\\ & \;\frac{\partial^{\alpha }s\left(x,y\right)}{\partial y^{\alpha }}\\ & \;≅\left(\frac{\alpha }{4}+\frac{\alpha^{2}}{8}\right)s\left(x,y+1\right)\\ & \;\;+\left(1-\frac{\alpha^{2}}{2}-\frac{\alpha^{3}}{8}\right)\times s\left(x,y\right)+\frac{1}{\Gamma \left(-\alpha \right)}\\ & \;\;\times \overset{n-2}{\underset{k=1}{\sum }}[\frac{\Gamma \left(k-\alpha +1\right)}{\left(k+1\right)!}\cdot \left(\frac{\alpha }{4}+\frac{\alpha^{2}}{8}\right)\;+\frac{\Gamma \left(k-\alpha \right)}{k!}\\ & \;\;\;\;\;\;\cdot \left(1-\frac{\alpha^{2}}{4}\right)+\frac{\Gamma \left(k-\alpha -1\right)}{\left(k-1\right)!}\cdot \left(-\frac{\alpha }{4}+\frac{\alpha^{2}}{8}\right)]\\ & \;\;\times s\left(x,y-k\right)+[\frac{\Gamma \left(n-\alpha -1\right)}{\left(n-1\right)!\Gamma \left(-\alpha \right)}\cdot \left(1-\frac{\alpha^{2}}{4}\right)\\ & \;\;\;\;\;\;\;\;\;+\frac{\Gamma \left(n-\alpha -2\right)}{\left(n-2\right)!\Gamma \left(-\alpha \right)}\cdot \left(-\frac{\alpha }{4}+\frac{\alpha^{2}}{8}\right)]\\ & \;\;\times s\left(x,y-n+1\right)+\frac{\Gamma \left(n-\alpha -1\right)}{\left(n-1\right)!\Gamma \left(-\alpha \right)}\\ & \;\;\cdot \left(-\frac{\alpha }{4}+\frac{\alpha^{2}}{8}\right)s\left(x,y-n\right). \end{array}$$

**Figure 2:**
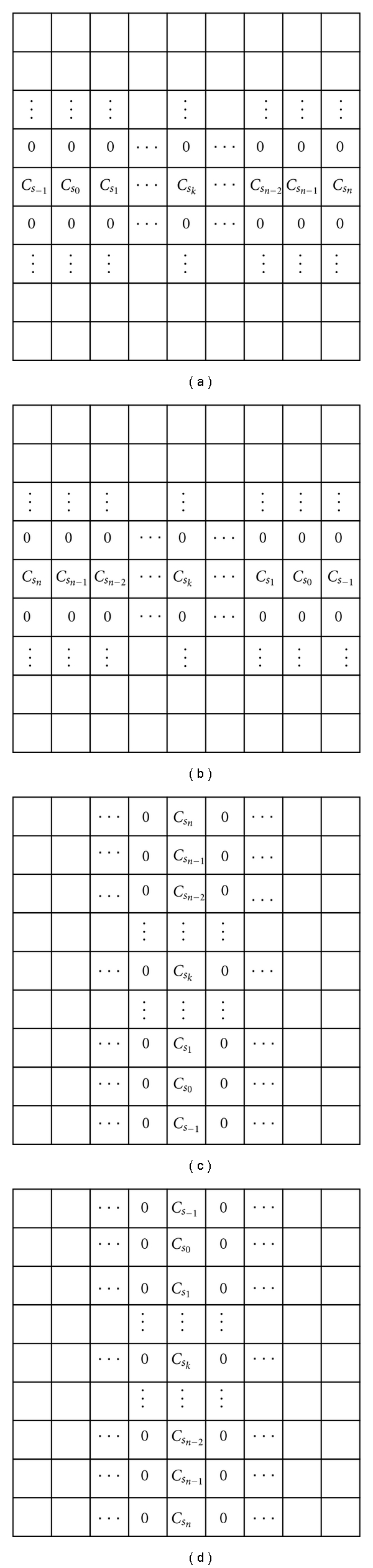
Masks of the four directions: (a) *D*_*x*+_^*α*^, (b) *D*_*x*−_^*α*^, (c) *D*_*y*+_^*α*^, and (d) *D*_*y*−_^*α*^.

For simplicity, we only use fractional order masks in four directions for calculation, including positive *x*- and *y*-coordinates and negative *x*- and *y*-axes. Let *D*_*x*+_^*α*^, *D*_*x*−_^*α*^, *D*_*y*+_^*α*^, and *D*_*y*−_^*α*^ denote the calculations in the four directions, as illustrated in Figure 2.

 The coefficients of the masks in Figure 2 are given below:

(16) $$\begin{array}{cc} C_{s_{-1}} & =\frac{\alpha }{4}+\frac{\alpha^{2}}{8}\\ C_{s_{0}} & =1-\frac{\alpha^{2}}{2}-\frac{\alpha^{3}}{8}\\ C_{s_{1}} & =-\frac{5\alpha }{4}+\frac{5\alpha^{3}}{16}+\frac{\alpha^{4}}{16}\\ \vdots & \\ C_{s_{k}} & =\frac{1}{\Gamma \left(-\alpha \right)}[\frac{\Gamma \left(k-\alpha +1\right)}{\left(k+1\right)!}\cdot \left(\frac{\alpha }{4}+\frac{\alpha^{2}}{8}\right)\;\\ & \;\;\;\;\;+\frac{\Gamma \left(k-\alpha \right)}{k!}\cdot \left(1-\frac{\alpha^{2}}{4}\right)\\ & \;\;\;\;\;\;+\frac{\Gamma \left(k-\alpha -1\right)}{\left(k-1\right)!}\cdot \left(-\frac{\alpha }{4}+\frac{\alpha^{2}}{8}\right)]\\ \vdots & \\ C_{s_{n-2}} & =\frac{1}{\Gamma \left(-\alpha \right)}[\frac{\Gamma \left(n-\alpha -1\right)}{\left(n-1\right)!}\cdot \left(\frac{\alpha }{4}+\frac{\alpha^{2}}{8}\right)\;\\ & \;\;\;\;\;+\frac{\Gamma \left(n-\alpha -2\right)}{\left(n-2\right)!}\cdot \left(1-\frac{\alpha^{2}}{4}\right)\\ & \;\;\;\;\;\;+\frac{\Gamma \left(n-\alpha -3\right)}{\left(n-3\right)!}\cdot \left(-\frac{\alpha }{4}+\frac{\alpha^{2}}{8}\right)]\\ C_{s_{n-1}} & =\frac{\Gamma \left(n-\alpha -1\right)}{\left(n-1\right)!\Gamma \left(-\alpha \right)}\cdot \left(1-\frac{\alpha^{2}}{4}\right)\\ & \;+\frac{\Gamma \left(n-\alpha -2\right)}{\left(n-2\right)!\Gamma \left(-\alpha \right)}\cdot \left(-\frac{\alpha }{4}+\frac{\alpha^{2}}{8}\right)\\ C_{s_{n}} & =\frac{\Gamma \left(n-\alpha -1\right)}{\left(n-1\right)!\Gamma \left(-\alpha \right)}\cdot \left(-\frac{\alpha }{4}+\frac{\alpha^{2}}{8}\right). \end{array}$$

Thus, we get a discrete representation of each item

(17) $$\begin{array}{cc} \Delta u & =u_{x}+u_{y},\\ u_{x} & =\text{min mod}\left(u_{x}^{c},\;\text{min mod}\left(2u_{x}^{b},2u_{x}^{f}\right)\right),\\ u_{y} & =\text{min mod}\left(u_{y}^{c},\;\text{min mod}\left(2u_{y}^{b},2u_{y}^{f}\right)\right),\\ {\left|\nabla^{\alpha }u\right|}^{-1} & =\frac{1}{\sqrt{\left({\left|D_{x+}^{\alpha }u\right|}^{2}+{\left|D_{y+}^{\alpha }u\right|}^{2}+\varepsilon \right)}},\\ \kappa^{\alpha } & =\nabla^{\alpha }\left(\frac{\nabla^{\alpha }u}{\left|\nabla^{\alpha }u\right|}\right)\\ & =D_{x-}^{\alpha }\left(\frac{D_{x+}^{\alpha }u}{\sqrt{\left({\left|D_{x+}^{\alpha }u\right|}^{2}+{\left|D_{y+}^{\alpha }u\right|}^{2}+\varepsilon \right)}}\right)\\ & \;+D_{y-}^{\alpha }\left(\frac{D_{y+}^{\alpha }u}{\sqrt{\left({\left|D_{x+}^{\alpha }u\right|}^{2}+{\left|D_{y+}^{\alpha }u\right|}^{2}+\varepsilon \right)}}\right) \end{array}$$

where *ε* is a small positive number to prevent dividing by zero, the superscript indexes *c*, *b*, and *f* denote central, backward, and forward differences, respectively, and the minmod function satisfies

(18) min mod (x,y)=sign (a)·max (0,min (|a|,b·sign (a))).

## 3. Results

 In this section, we present the experimental results of our FCDD model compared with the LI and TV models. 

 As there is no quantitative method to measure the performance of CT MAR [11], we apply the peak-signal to-noise ratio (PSNR), which is commonly used in image inpainting [26], as the available criterion

(19) $$\text{PSNR}=10\times \text{lo}{\text{g}}_{10}\left(\frac{255^{2}}{{||u-u_{0}||}_{2}^{2}}\right).$$

Here, *u* is the image after inpainting and *u*_0_ is the original image. The greater the value of the PSNR is, the better is the performance. PSNR is usually used to measure the similarity between an inpainted image and a real image, and as such it is a suitable reference. 

 Several concrete steps are followed in our method. First, because in this paper we focus on the inpainting algorithm, for simplicity, we only use the threshold method to extract the metal region. It is easy to say that a more accurate segmentation algorithm would enhance the performance of MAR. Second, we locate the corresponding metal regions in the projection data set. Third, we employ the FCDD algorithm to inpaint the metal regions. Finally, we reconstruct the image from the inpainted sinogram and insert the metal regions.

 In the first experiment, while dealing with multiple metals, we compared the performance of FCDD against other methods. Five metal regions with much higher attenuation were added into the Shepp-Logan (S-L) phantom (256 × 256) to simulate the metal artifacts. The parameters are given in Table 1.

**Figure 3:**
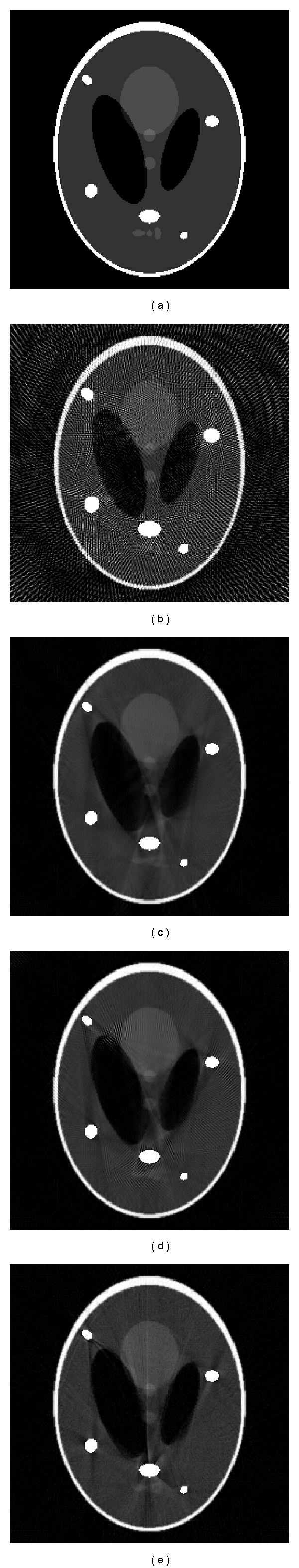
Comparison of different sinogram inpainting methods with multiple metal regions: (a) phantom with five metal regions, (b) filtered back projection with metal artifacts, (c) filtered back projection after LI (PSNR = 26.4205), (d) filtered back projection after TV (PSNR = 25.9497), and (e) filtered back projection after FCDD (PSNR = 26.4399).

**Figure 4:**
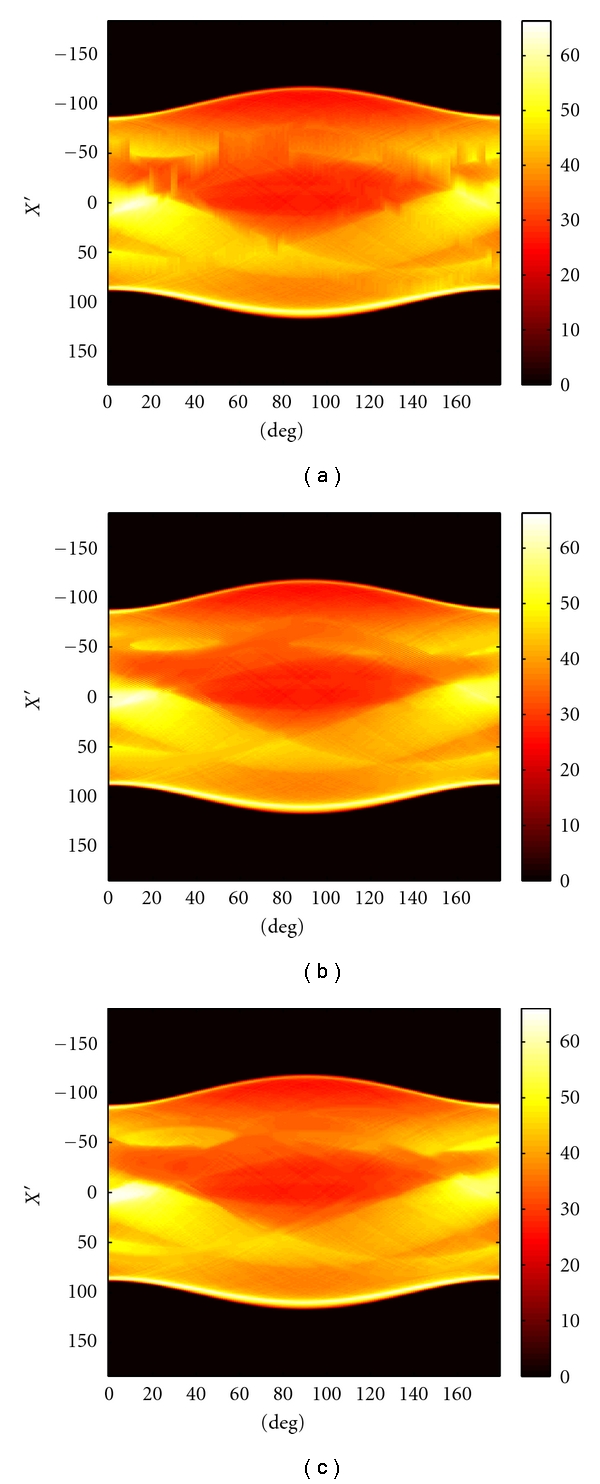
Sinogram inpainting results for different methods with multiple metal regions: (a) sinogram after LI, (b) sinogram after TV, and (c) sinogram after FCDD.

Figure 3 shows the results of the different algorithms, where Figure 3(a) gives the original phantom with a metal region while Figure 3(b) shows the phantom reconstructed from the projection data and containing severe metal artifacts. Figures 3(c) to 3(e) illustrate the results of LI, TV, and FCDD, respectively. In these figures, we can see that metal artifacts are suppressed to different degrees. Compared with LI and TV, FCDD achieves a better visual effect. The structure information (edges and shapes) in Figure 3(e) (*α* = 1.8) is the clearest. In particular, the shapes of the virtual organs are almost maintained, except that the TV method causes an artificial effect. Near the metal regions, the staircase effect is marked. The results of FCDD show the best performance with the highest PSNR. To explain the reason for this, we also give the sinograms after inpainting with these three methods in Figure 4. 

 When there are multiple metal regions, the gaps to be inpainted are much wider than a single metal region and there is much more missing information that needs to be interpolated. The classic interpolation methods only use the information in the same column for interpolation. The useful information is too scant to obtain an accurate result, as shown in Figure 4(a). As the TV and FCDD methods are 2D inpainting methods, they make use of not only the information in the columns, but also that in the rows and thus they obtain better results. However, TV has a flaw in dealing with wide regions, and the staircase effect occurs. The reason for this is that the order of TV is 2 and to obtain an accurate result, the order must be greater than 2 [27] but less than 4 [28]. In this experiment, we set *α* = 1.8 in FCDD, which means that the order approximates to 3 and thus the visual effects of inpainting sinograms of FCDD are superior.

**Figure 5:**
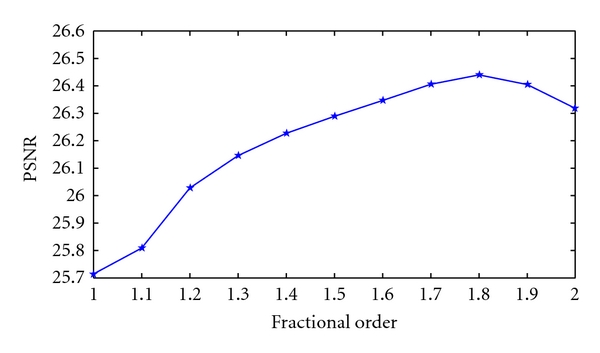
Variations in PSNR with fractional order.

Figure 5 gives the variation of the PSNR value with the order of the algorithm. If *α* = 1, the model is the same as in [22]. As the order increases, the PSNR value also increases. If *α* ≈ 1.8, the PSNR peaks and subsequently declines. 

**Figure 6:**
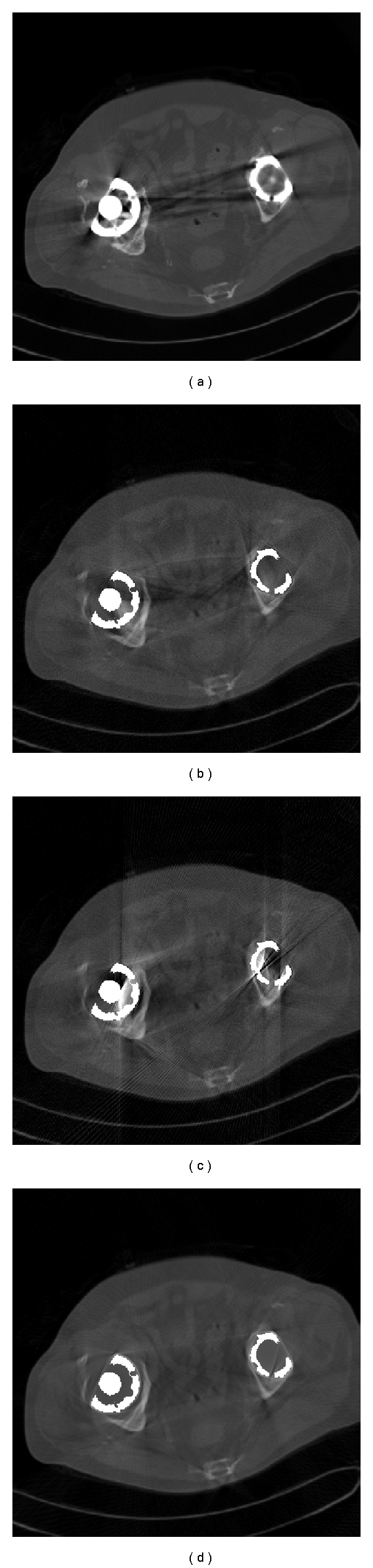
Comparison of different methods dealing with a clinical case: (a) original image with metal artifacts, (b) FBP after LI, (c) FBP after TV, and (d) FBP after FCDD.

Figure 6 shows the clinical case of a patient with metal implants in both femurs. In this experiment, we do not know the original image, so we cannot choose *α* according to the PSNR. Based on the analysis in the previous experiment, we approximately set *α* = 1.8.  Figure 6(a) shows the original image with dark, board streaks radiating from the metals. Figures 6(b) to 6(d) illustrate the reduction in the most severe artifacts using LI, TV, and FCDD, respectively. The dark streaks are not adequately suppressed in Figures 6(b) and 6(c), while fictitious artifacts caused by TV are visible in Figure 6(c). These disturbing artifacts are reduced to a large degree in Figure 6(d). Structural information previously invisible because of artifacts or the incomplete correction becomes visible.

## 4. Conclusion

 A new MAR algorithm has been proposed based on the classic TV inpainting model. We replaced the conditional conductivity coefficient for TV with a new fractional-order curvature, and in comparison with linear interpolation and TV, our method obtained better quantitative results and visual effects.

 To achieve the best performance for different images, the fractional order also needs to be different. Thus, future work will focus on the adaptive selection of the order of the algorithm.

## Figures and Tables

**Table 1:** Parameters of the modified S-L phantom with five metal regions: *τ* is the attenuation coefficient; *A*, *B* are the lengths of the semiaxes of each ellipse; *x*, *y* are the central coordinates of each ellipse; *θ* is the rotation angle (in degrees).

No.	*τ*	*A*	*B*	*x*	*y*	*θ*
1	1.0	0.920	0.6900	0	0	90
2	−0.8	0.874	0.6624	0	−0.0184	90
3	−0.2	0.310	0.1100	0.22	0	72
4	−0.2	0.410	0.1600	−0.22	0	108
5	0.1	0.250	0.2100	0	0.3500	90
6	0.1	0.046	0.0460	0	0.1000	0
7	0.1	0.046	0.0460	0	−0.1000	0
8	0.1	0.046	0.0230	−0.08	−0.6050	0
9	0.1	0.023	0.0230	0	−0.6050	0
10	0.1	0.046	0.0230	0.06	−0.6050	90
11	30.0	0.075	0.0500	0	−0.4800	0
12	25.0	0.030	0.0250	0.25	−0.6200	45
13	30.0	0.040	0.0300	−0.45	0.5000	135
14	30.0	0.050	0.0400	0.45	0.2000	0
15	30.0	0.050	0.0450	−0.42	−0.3000	72
